# 2D Au Nanosphere Arrays/PVA-PBA-Modified-Hydrogel Composite Film for Glucose Detection with Strong Diffraction Intensity and Linear Response

**DOI:** 10.3390/nano9020140

**Published:** 2019-01-22

**Authors:** Wenjuan Li, Junhuai Xiang, Dandan Men, Honghua Zhang

**Affiliations:** Jiangxi Key Laboratory of Surface Engineering, Jiangxi Science and Technology Normal University, Nanchang 330013, China; LWenjuan27@126.com (W.L.); xiangjunhuai@163.com (J.X.)

**Keywords:** 2D Au nanosphere arrays, PVA-PBA-modified-hydrogel composite film, glucose detection, diffraction intensity, linear response

## Abstract

A novel glucose sensor was reported that consisted of two-dimensional (2D) Au nanosphere arrays and glucose-responsive hydrogel film. This sensor exhibited an intense diffraction signal and an obvious diffraction color on a quartz slide due to the strong diffraction intensity of the Au nanosphere arrays. Thus, glucose was detected via the variation of diffraction wavelength and diffraction color, without a high reflective mirror. In addition, by introducing poly(vinyl alcohol) (PVA) to crosslink the phenylboronic acid (PBA)-modified hydrogel film, the diffraction wavelength of the 2D Au nanosphere arrays/hydrogel composite film shifted in the same direction in high ionic strength condition. In particular, it showed a nearly linear red-shift when the glucose concentration increased from 0 mM to 20 mM. Moreover, this glucose sensor displayed good reproducibility. The nearly linear response and good reproducibility were highly helpful for improving practical application of this glucose sensor.

## 1. Introduction

Diabetes, one of the largest health concerns, has been diagnosed through monitoring the concentration of blood glucose [1,2]. Real-time monitoring and controlling glucose concentration are important for making optimal therapeutic decisions to improve the lives of diabetic patients [3,4]. So far, various optical [5,6,7,8,9,10,11], colorimetric [12,13], and electrochemical [14,15] sensors have been developed for detecting glucose. Among these, the optical sensors based on photonic crystals (PCs) have received considerable attention because they can detect target analytes with the naked eye, without sophisticated equipment and professional operators [16,17,18,19,20,21,22,23,24,25,26]. In particular, with the composites of hydrogel and PCs as sensors, interactions between the hydrogel matrix and an analyte result in the volumetric change of the hydrogel, leading to a change of periodicity of the embedded PCs. As the periodicity of PCs either increases or decreases, the diffraction wavelengths would red or blue shift, accompanied by a series of color changes, which could be detected with the naked eye. In this case, the analyte concentration could be visually estimated by diffraction color and quantitatively reported through the shift of the diffraction wavelength [16,17,18,19,20]. For example, a hydrogen fluoride (HF) acid sensor was prepared by embedding the PCs in the HF-sensitive hydrogel film [27]. In this HF acid sensor, as the HF acid concentration increased from 0 to 20 Mm, the diffraction wavelength red-shifted from 557 to 763 nm.

Phenylboronic acids (PBAs) can bind with glucose to form complexes, leading to volumetric change of hydrogel [28,29,30,31,32]. Therefore, the glucose-responsive hydrogels could be synthesized by functionalizing hydrogel with PBAs. Additionally, the PBA-modified hydrogels possess good operational stability [4]. In the past decade, three-dimensional (3D) colloidal crystalline arrays as the PCs, were embedded into the PBA-modified hydrogel matrix to sense glucose [4,6,8,9]. However, in this method, the preparation process of 3D PCs is time-consuming, and the functionalizing process of the hydrogel matrix is relatively complex [32]. For example, more than one week was needed for preparing the 3D arrays [31,32]. Compared with 3D arrays, the preparation of two-dimensional (2D) arrays is easier and faster. For instance, 2D arrays of polystyrene (PS) spheres with an area ca. of 15 cm^2^ were produced within ca. 30 s by the air/water interface self-assembly approach. Therefore, visual sensors of 2D arrays attracted significant attention [32,33,34]. A glucose visual sensor was developed by embedding the 2D PS colloidal arrays in a PBA-functionalized hydrogel film [32]. In this glucose sensor, besides the rapid preparation process, only one modification step was needed to couple PBAs to the hydrogel matrix. However, both the diffraction measurement and color identification of the 2D array glucose sensor relied on a high reflective mirror due to low reflectivity of PS. This influenced their practical application in visual detection. Therefore, it was desirable to develop a 2D array glucose sensor with a high diffraction intensity to easily achieve visual and instrumental monitoring.

In this work, 2D Au nanosphere arrays were embedded in a PBA-modified hydrogel matrix to prepare the glucose sensor. The strong diffraction intensity of Au nanosphere arrays allowed the 2D Au nanosphere arrays/hydrogel composite films to exhibit an obvious diffraction signal and diffraction color on the quartz slide. Thus, the concentration of glucose was detected by changes of diffraction wavelength and color, without a high reflective mirror. Most importantly, after introducing poly(vinyl alcohol) (PVA) to crosslink the PBA-modified hydrogel film, the diffraction wavelength of the composite film shifted in the same direction (red-shift) in high ionic strength condition. In particular, it showed a nearly linear red-shift when the glucose concentration increased from 0 mM to 20 mM, which is highly helpful for improving practical application of this glucose sensor.

## 2. Materials and Methods

### 2.1. Materials

Polystyrene (PS) nanospheres with a diameter of 500 nm in aqueous suspension (2.5 wt%) were purchased from Alfa Aesar Corporation (Shanghai, China). Acrylamide (AAm), dimethyl sulfoxide (DMSO), glucose, sodium hydroxide, and poly(vinyl alcohol) (PVA) were obtained from Sinopharm Chemical Reagent Co., Ltd. (Shanghai, China). *N*,*N*′-methylenebisacrylamide (MBAAm) was purchased from Shanghai Aladdin Bio-Chem Technology Co., Ltd. (Shanghai, China). 2-hydro-xy-1-[4-(2-hydroxyethoxy)-phenyl]-2-methyl-1-propanone (Irgacure 2959) was obtained from Tianjin Heowns Biochemical Technology Co., Ltd. (Tianjin, China), and 3-acrylamidophenylboronic acid (PBA) was purchased from Beijing HWRK Chem Co., Ltd. (Beijing, China). 2-(cyclohexylamino)ethanesulfonic acid (CHES) were obtained from Adamas (Shanghai, China). They were directly used without further purification. Water (18.2 MΩ·cm) was obtained from an ultrafilter system (Milli-Q, Millipore, Marlborough, MA, USA).

### 2.2. Preparation of the 2D Au Nanosphere Arrays

**Figure 1:**
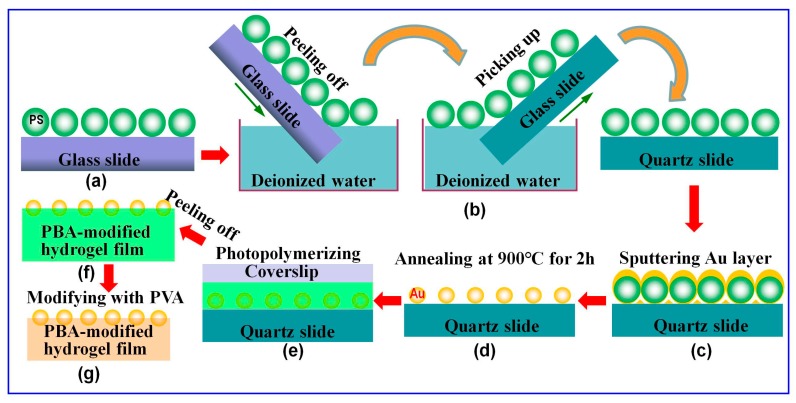
Preparation of the two-dimensional (2D) Au nanosphere arrays/glucose-sensitive hydrogel composite film: (**a**) 2D polystyrene (PS) colloidal monolayer arrays were fabricated on a clean glass slide by an air/water interfacial self-assembly method; (**b**) transferring the 2D PS monolayer arrays to a quartz slide; (**c**) depositing a layer of Au film on the 2D PS colloidal monolayer arrays; (**d**) annealing at 900 °C for 2 h; (**e**) pouring reaction solution into the 2D Au nanosphere arrays and subsequently photopolymerizing with UV light; (**f**) a free-standing 2D Au nanosphere arrays/hydrogel composite film was obtained by peeling it from the substrate; and (**g**) immersing as-prepared composite film into a poly(vinyl alcohol) PVA aqueous solution to crosslink it with PVA.

The 2D Au nanospheres arrays were prepared on a quartz substrate by using PS colloidal monolayer arrays as initial templates, followed by depositing the Au layer and subsequent annealing, as reported previously [27,35,36,37]. Briefly, the PS colloidal monolayer arrays were fabricated on a glass slide by self-assembly at air/water interface (Figure 1a). Then, the dried PS colloidal monolayer arrays on a glass slide were immersed slantwise into water in a beaker. In this process, the PS colloidal monolayer arrays were peeled off from their substrate (the glass slide) and floated on the surface of the water. The PS colloidal monolayer arrays located on the surface of the water were transferred onto a quartz slide simply by picking it up with the new substrate, as shown in Figure 1b [38]. Subsequently, a layer of Au film was deposited on the top of the as-prepared PS microsphere templates by an ion-beam coater in a vacuum of 1 × 10^−1^ mbar with a deposition time of 3 min and current of 20 mA (Figure 1c). Finally, the prepared 2D PS colloidal crystals with Au film were annealed at 900 °C for 2 h. Through these processes, ordered 2D Au nanosphere arrays with a hexagonal non-close-packed (HNCP) arrangement were fabricated (Figure 1d).

### 2.3. Preparation of the 2D Au Nanosphere Arrays/Glucose-Sensitive Hydrogel Composite Film

Briefly, reaction solution was obtained by mixing 0.350 g AAm, 0.008 g MBAAm, 200 µL APBA solution (50% (*w*/*v*) in DMSO) and 35 µL Irgacure 2959 solution (33% (*w*/*v*) in 1 mL DMSO) with 3 mL deionized water to form a transparent solution. Then, 100 µL reaction solution was poured into the 2D Au nanosphere arrays, followed by carefully layering a coverslip on the solution. Subsequently, the reaction solution was polymerized by initiating with a 365 nm UV Lamp (16 w) for ca. 30 min at room temperature (Figure 1e). The resulting 2D Au nanosphere arrays/PBA-modified hydrogel composite films were peeled from the substrate and coverslip, and then rinsed with a lot of water (Figure 1f). Finally, the composite films were immersed in a PVA aqueous solution (1.25 wt%) for 24 h to obtain the PVA-PBA-modified hydrogel composite film (Figure 1g).

### 2.4. Preparation of Buffer Solution

For the low ionic strength buffer solution at pH 9.0, 1.044 g CHES was dissolved in 450 mL of deionized water, followed by adding 1 M NaOH to adjust the pH to 9.0 and dilution to 500 mL with deionized water. A high ionic strength buffer solution (pH 9, ionic strength ca. 150 mM) was made by mixing 1.558 g CHES and 0.719 g NaCl in 50 mL deionized water, followed by titration to pH 9.0 with 1 M NaOH and dilution to 100 mL with deionized water [8,32]. Glucose was added to yield concentrations ranging from 2 mM to 80 mM.

### 2.5. Characterization

The morphologies of the as-prepared samples were characterized by a field-emission scanning electron microscope (FESEM, Sirion200, Hillsboro, OR, USA) after sputter-coating a thin layer of Au. The diffraction spectra of the 2D Au nanosphere array/hydrogel composite film response to different glucose concentrations were obtained at a specific angle by using an Idea Optics PG2000-Pro-EX spectrometer (Shanghai, China), R1-A-UV support and Halogen and Deuterium light source. In the process of diffraction measurements, the 2D Au nanosphere arrays/hydrogel composite films were placed on quartz slides. In order to make the diffraction wavelength of the 2D Au nanosphere arrays/hydrogel composite film in the visible region, we adjusted the angle between the light source and the normal to 2D array/hydrogel composite films to 23° in low ionic strength buffer solution and 32° in high ionic strength buffer solution, respectively. The 2D Au nanosphere arrays/hydrogel composite films were equilibrated overnight before diffraction measurements. Their photographs were taken using a digital camera (Sony, Cyber-shot DSC-N2, Tokyo, Japan). Additionally, these composite films were placed on a quartz slide during the diffraction measurement and photographing. The Fourier Transform Infrared (FTIR) spectra were collected using an Intelligent Fourier infrared Raman spectrometer (NEXUS, Thermo Nicolet Corporation, Madison, WI, USA).

## 3. Results and Discussion

### 3.1. Sensing Glucose by the 2D Au Nanosphere Arrays/PBA-Modified Hydrogel Composite Film in Low Ionic Strength Buffer Solution

**Figure 2:**
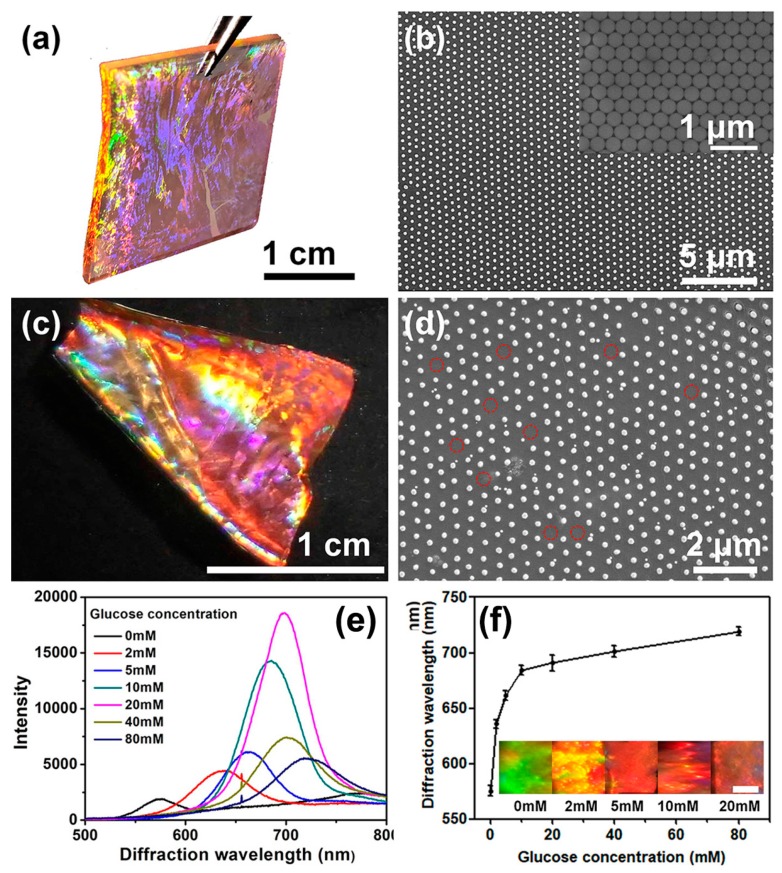
Photograph (**a**) and SEM image (**b**) of the 2D Au nanosphere arrays (Inset: SEM image of the PS colloidal monolayer arrays template); photograph (**c**) and SEM image (**d**) of 2D Au nanosphere arrays/PBA-modified hydrogel composite film; (**e**) representative diffraction spectra of the 2D Au nanosphere arrays/PBA-modified hydrogel composite films in low ionic strength buffer solution at different glucose concentrations; and (**f**) glucose concentration dependence of the diffraction wavelength of the composite films (Inset: photographs of 2D Au nanosphere arrays/PBA-modified hydrogel composite films at different glucose concentrations. The scale bar is 0.15 cm). Error bars represent standard deviation (SD) of four samples.

Highly ordered 2D Au nanosphere arrays with an area of 2 cm × 2 cm were prepared using PS colloidal monolayer arrays as templates, followed by depositing the Au layer and annealing treatment, as described in our previous report [37]. Figure 2a shows the digital photograph of the 2D Au nanosphere arrays. It can be seen the 2D Au nanosphere array displayed a bright iridescent color, resulting from the strong diffraction of Au nanosphere arrays with an ordered array structure. This conclusion was further supported by the SEM image, as shown in Figure 2b. One can find that a highly ordered HNCP arrangement on the substrate was observed, and the Au nanoparticles presented a near-spherical morphology. This indicated that the ordered array structure of the PS colloidal monolayer template was maintained after depositing Au layer and annealing process. The inset of Figure 2b shows the array structure of the PS colloidal monolayer arrays. The highly ordered 2D Au nanosphere arrays with HNCP arrangement were transferred to hydrogel film by in-situ photopolymerization. In this process, 100 µL reaction solution (AAm as a monomer, PBA as a functional monomer, MBAAm as a cross-linker, and Irgacure 2959 as an initiator) was poured into the substrate and subsequently potopolymerized. The resultant 2D Au nanosphere array/PBA-modified hydrogel composite film also exhibited bright color (Figure 2c), similar to the 2D Au nanosphere arrays. Its SEM image (Figure 2d) demonstrates that the ordered structure of Au nanosphere arrays was not destroyed during the polymerization and transfer process. However, further observation revealed that some Au nanospheres were not transferred to hydrogel film (as shown in the red circles of Figure 2d), but this made a negligible influence to the diffraction intensity.

PBA is widely used for glucose-responsive sensors due to high operational stability [4]. In this work, the PBA-modified hydrogel film as a glucose sensor was prepared by copolymerizing AAm and PBA. Figure 2e presents the representative diffraction spectra of as-prepared 2D Au nanosphere array/PBA-modified hydrogel composite film in low ionic strength buffer solution containing various glucose concentrations. As illustrated in Figure 2e, although the hydrogel films were placed on a quartz slide during measuring process, they retained intense diffraction peaks. The reason was attributed to the large scattering cross section of Au nanospheres [35].

Moreover, it can be found the diffraction peak gradually red shifted with increasing glucose concentration, as shown in Figure 2e. The dependence of the diffraction peak position of this sensor with different glucose concentrations is shown in Figure 2f. With an increase of glucose concentration, a clear red-shift trend of the diffraction peak position was observed. Meanwhile, the shift of diffraction wavelength accompanied with a series obvious color variation (the inset of Figure 2f), which can be recognized by the naked eye. The glucose-induced diffraction red shift results from formation of boronate anions by covalently binding glucose to PBA to form 1:1 PBA-glucose complex in low ionic strength buffer solution [3,6,8], as shown in Figure S1 (Supporting Information). The formed boronate anions would result in a Donnan potential that gives rise to an osmotic pressure, thereby, actuates swelling of the hydrogel and red-shift of the diffraction wavelength.

### 3.2. Sensing Glucose by the 2D Au Nanosphere Arrays/PBA-Modified Hydrogel Composite Film in High Ionic Strength Buffer Solution

**Figure 3:**
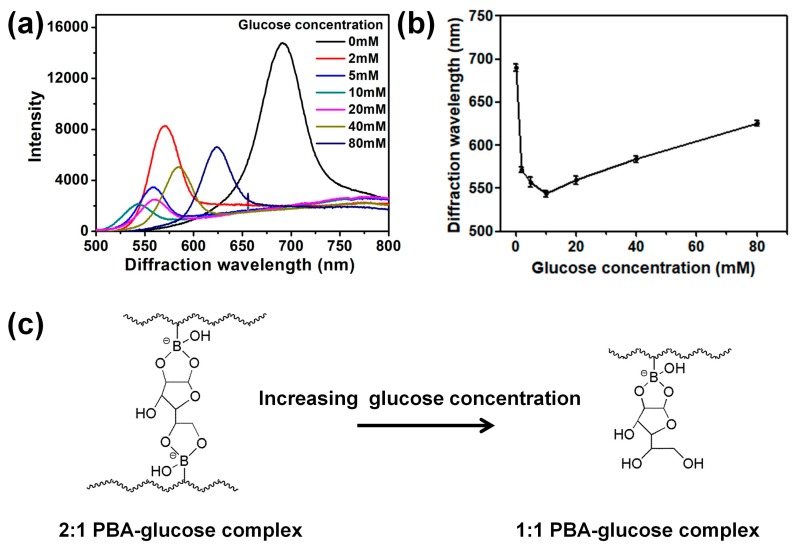
(**a**) Representative diffraction spectra of 2D Au nanosphere arrays/PBA-modified hydrogel composite films in high ionic strength buffer solution at different glucose concentrations; (**b**) glucose concentration dependence of the diffraction wavelength of the composite films; and (**c**) interactions of PBA-modified hydrogel with the furanose form of glucose in high ionic strength buffer solution with increasing glucose concentration. Error bars represent standard deviation (SD) of four samples.

Here, the response of 2D Au nanosphere array/PBA-modified hydrogel film to glucose was investigated in a high ionic strength buffer solution (ca. 150 mM), which is close to the physiological ionic strength. As shown in Figure 3a, the composite films show a blue-shift of diffraction wavelength with increasing the concentration up to 10 mM. However, as the glucose concentration further increased to 20 mM, a red-shift was observed, in agreement with previously reported works [4,6,30]. Moreover, the dependence of the diffraction peak position of 2D Au nanosphere arrays/PBA-modified hydrogel film on different glucose concentrations in high ionic strength buffer solution is shown in Figure 3b. It clearly displays that the position of diffraction peak decreased firstly and then increased with increasing glucose concentration.

At low glucose concentrations, the blue-shift of the diffraction wavelength derived from formation of 2:1 PBA-glucose complex, which generated additional crosslinking in the hydrogel matrix (Figure 3c) [4,6,32]. The additional crosslinking degree would lead the hydrogel to shrink, thus inducing a blue-shift of the diffraction wavelength [4,6,30]. The formation of 2:1 PBA-glucose complex may be ascribed to the relatively close distance of neighboring PBA groups where the hydrogel matrix would be in the shrinking state in high ionic strength solution [32]. Conversely, in low ionic strength solution, the hydrogel carrier would be in a swelling state. In this case, the distance between two neighboring PBA groups is too far to form 2:1 PBA-glucose complex. Moreover, in order to gain an insight into the interaction of glucose with the PBA-modified hydrogel film in different ionic strength buffer solution, the FTIR spectra of the dried PBA-modified hydrogel composite film were measured after immersing in high and low ionic strength buffer solution with a glucose concentration of 5 mM, respectively. In the FTIR spectra (Figure S2, Supporting Information), the peak appearing at 1260 cm^−1^ was assigned to the B-OH vibration [39]. Its signal intensity in ionic strength buffer solution was weaker than that in low ionic strength buffer solution. This may be attributed to more B-OH react with glucose due to the formation of 2:1 PBA-glucose complex in ionic strength buffer solution.

However, at high glucose concentrations, the formation of 2:1 PBA-glucose complex would be broken into two charged 1:1 PBA-glucose complexes (Figure 3c). As this happens, the crosslinking degree of hydrogel decreases, but the ionization degree increases [4,6,30]. The increasing ionization degree would give rise to an expansion in hydrogel volume and a red-shift of the diffraction wavelength. The conversion between 1:1 and 2:1 PBA-glucose complex would cause interference for practical application because this sensor may display the same diffraction wavelength at different glucose concentrations. For this sensor, the diffraction peaks were almost in the same positions when the glucose concentrations are 5 mM (557 nm) and 20 mM (559 nm), respectively. In this case, it would be difficult to identify the different glucose concentrations by the shift of diffraction wavelength. In order to solve this problem, the 2D Au nanosphere array/PVA-PBA-modified hydrogel composite film was prepared.

### 3.3. Sensing Glucose by the 2D Au Nanosphere Array/PVA-PBA-Modified Hydrogel Composite Film in High Ionic Strength Buffer Solution

**Figure 4:**
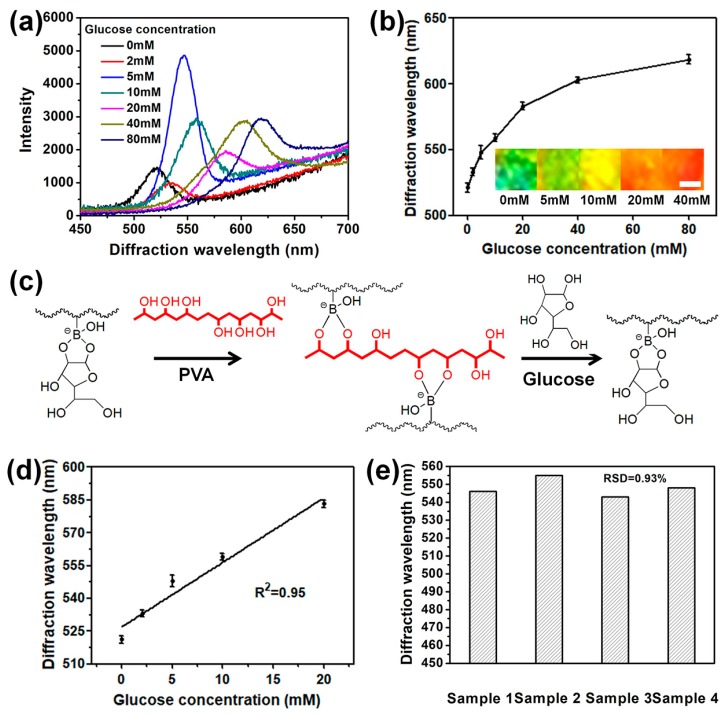
(**a**) Representative diffraction spectra of 2D Au nanosphere arrays/PVA-PBA-modified hydrogel composite films in high ionic strength buffer solution at different glucose concentrations; (**b**) glucose concentration dependence of the diffraction wavelength of the composite films (Inset: photographs of 2D Au nanosphere arrays/PVA-PBA-modified hydrogel composite films at different glucose concentrations, the scale bar is 0.15 cm); (**c**) and interaction of PBA-modified hydrogel with the PVA and interaction of the PVA-PBA-modified hydrogel composite film with glucose in high ionic strength buffer solution. (**d**) Diffraction peak positions of the 2D Au nanosphere arrays/PVA-PBA-modified hydrogel composite films at the glucose concentration from 0 mM to 20 mM. The line is linear fit. Error bars represent standard deviation (SD) of four samples. (**e**) Diffraction wavelength of different samples at the glucose concentration of 5 mM.

It is known that PBAs have lower affinities to 1,3-diols than 1,2-diols. Therefore, the preformed 1,3-diol-PBA complexes can be displaced by 1,2-diols molecules to form 1,2-diol-PBA complexes [4]. According to this mechanism, Braun’s group synthesized a glucose sensor based on 3D arrays that shows linear response to glucose under physiological conditions. However, in their work, the preparation process of the 3D arrays was time-consuming. Furthermore, relatively complex modification steps were required to couple the PBA to the hydrogel matrix. In this work, 2D Au nanosphere arrays/PVA-PBA-modified hydrogel composite film as a glucose sensor was prepared by a relatively simple preparation method. For instance, PBA was directly introduced into the hydrogel matrix by in-situ copolymerization of AAm and APBA. Then, the preformed 2D Au nanosphere arrays/PBA-modified hydrogel composite films were immersed in PVA aqueous solution to crosslink the hydrogel with PVA. Figure 4a shows representative diffraction spectra of the 2D Au nanosphere array/PVA-PBA-modified hydrogel composite film at different glucose concentrations in CHES buffer solution with ionic strength of ca. 150 mM. Compared with Figure 3a,b, the phenomenon that blue-shift first and then red-shift disappeared, and the diffraction peaks were always red-shift with increasing glucose concentration. In addition, from Figure 4b, an obvious shift trend at the same direction was observed with increasing glucose concentration. Moreover, the diffraction color of the sensors changed from green to yellow-green, yellow, orange, and then red (inset in Figure 4b). For this phenomenon, the following mechanism has been proposed. Before sensing glucose, PVA was bonded with the immobilized PBA, resulting in additional crosslinking that reduced the hydrogel volume, as shown in Figure 4c. When exposed to glucose, preformed PVA-PBA complexes were dissociated into 1:1 glucose-PBA complexes because glucose was able to form a stronger complex with PBA than PVA. The formation of 1:1 glucose-PBA complexes leads the hydrogel only to swell, accompanied with red-shift of diffraction peaks.

**Figure 5:**
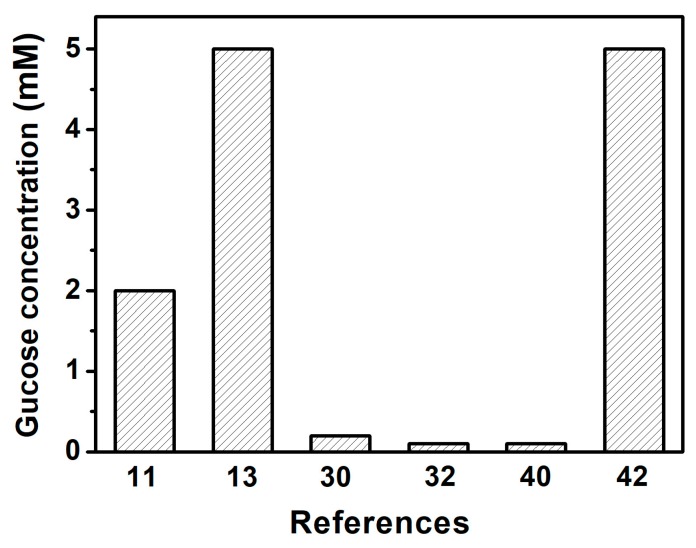
The reported detected lowest glucose concentration based on photonic crystals (PCs) glucose sensors in the References.

Further research suggests that the response of this 2D Au nanosphere arrays/PVA-PBA-modified hydrogel composite film was in close linear correlation to glucose concentration when it ranged from 0 mM to 20 mM (R^2^ = 0.95), as shown in Figure 4d. Additionally, the reproducibility of the 2D Au nanosphere arrays/PVA-PBA-modified hydrogel composite film as a glucose sensor was studied. Figure 4e shows the diffraction wavelength of four samples at the glucose concentration of 5 mM. The results demonstrated that the glucose sensor had higher reproducibility. Moreover, the sensitivity of this glucose sensor was also explored, and it exhibited significant red-shift when the glucose concentration was above 2 mM. Although the minimum detectable concentration in this work was relatively high, compared with the results of other previously published works (Figure 5) [40,41,42], it still could satisfy the detecting requirements of blood glucose for diabetic patients because normal-fasting blood sugar levels range from approximately 4 to 6 mM in the human body [2,11,40,41]. The yielded 2D glucose sensor with a linear response to glucose and good reproducibility under physiological condition would improve the practical application of the 2D array hydrogel materials.

## 4. Conclusions

In this research, a novel glucose-responsive 2D arrays/hydrogel composite film with high diffraction intensity was developed by embedding the 2D Au nanosphere arrays into the PBA-modified hydrogel film. This composite film displayed an obvious diffraction signal and diffraction color on the quartz slide. Therefore, glucose could be quantitatively detected through the position of the diffraction peak and visually estimated by the diffraction color on the transparent substrate. Additionally, by introducing PVA to PBA-modified hydrogel matrix, the diffraction wavelength of the resulting 2D Au nanosphere arrays/PVA-PBA-modified hydrogel sensor shifted in the same direction (red-shift) in high ionic strength conditions. In particular, it showed nearly linear red-shift when the glucose concentration increased from 0 mM to 20 mM, which was useful in improving the practical application of the glucose sensor.

## Supplementary Materials

The following are available online at http://www.mdpi.com/2079-4991/9/2/140/s1, Figure S1: Chemical structure of 1:1 Phenylboronic acid (PBA)-glucose complex, Figure S2: FTIR spectra of the dried PBA-modified hydrogel composite film after immersing in high (I) and low ionic strength buffer solution (II) with a glucose concentration of 5 mM, and dried PBA-modified hydrogel composite film without binding with glucose (III).
