# Supplementary material for: 2D Au Nanosphere Arrays/PVA-PBA-Modified-Hydrogel Composite Film for Glucose Detection with Strong Diffraction Intensity and Linear Response
Source: Nanomaterials (Basel). 2019 Jan 22;9(2):140. doi: 10.3390/nano9020140 (PMC6409588; doi:10.3390/nano9020140)
Supplement: Supplementary file 1 [file nanomaterials-09-00140-s001.pdf]

Supporting Information

# 2D Au Nanosphere Arrays/PVA-PBA-Modified-Hydrogel Composite Film for Glucose Detection with Strong Diffraction Intensity and Linear Response

**Wenjuan Li, Junhuai Xiang, Dandan Men * and Honghua Zhang ***

Jiangxi Key Laboratory of Surface Engineering, Jiangxi Science and Technology Normal University, Nanchang 330013, China; LWenjuan27@126.com (W.L.); xiangjunhuai@163.com (J.X.)
* Correspondence: mendandan1999@126.com (D.M.); zhanghonghua@impcas.ac.cn (H.Z.)

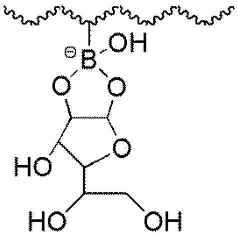


**Figure S1.** Chemical structure of 1:1 Phenylboronic acid (PBA)-glucose complex.

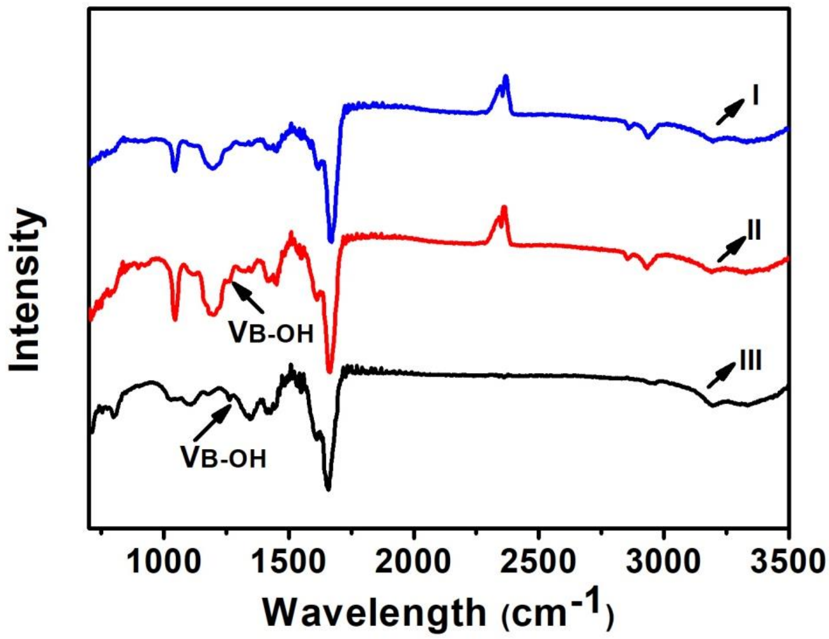


**Figure S2.** FTIR spectra of the dried PBA-modified hydrogel composite film after immersing in high (I) and low ionic strength buffer solution (II) with a glucose concentration of 5 mM, and dried PBA-modified hydrogel composite film without binding with glucose (III).
